# Social distancing, community stigma, and implications for psychological distress in the aftermath of Ebola virus disease

**DOI:** 10.1371/journal.pone.0276790

**Published:** 2022-11-02

**Authors:** Thomas M. Crea, K. Megan Collier, Elizabeth K. Klein, Stephen Sevalie, Bailah Molleh, Yusuf Kabba, Abdulai Kargbo, Joseph Bangura, Henry Gbettu, Stewart Simms, Clara O’Leary, Stacy Drury, John S. Schieffelin, Theresa S. Betancourt

**Affiliations:** 1 School of Social Work, Boston College, Chestnut Hill, Massachusetts, United States of America; 2 Sustainable Health Systems, Freetown, Sierra Leone; 3 Sierra Leone Association of Ebola Survivors, Freetown, Sierra Leone; 4 Caritas Freetown, Freetown, Sierra Leone; 5 School of Medicine, Tulane University, New Orleans, Louisiana, United States of America; Njala University, SIERRA LEONE

## Abstract

**Background:**

The 2013–2016 Ebola virus disease (EVD) epidemic resulted in more infections and deaths than all prior outbreaks in the 40-year history of this virus combined. This study examines how experiences of EVD infection, and preventive measures such as social distancing, were linked to experiences of stigma and social exclusion among those reintegrating into their communities.

**Methods:**

Key informant interviews (*n* = 42) and focus group discussions (*n* = 27) were conducted in districts with a high prevalence of EVD and representing geographical and ethnic diversity (*n* = 228 participants). The final sample was composed of adults (52%) and children (48%) who were EVD-infected (46%) and -affected (42%) individuals, and community leaders (12%). Data were coded using a Grounded Theory approach informed by Thematic Content Analysis, and analyzed using NVivo. Interrater reliability was high, with Cohen’s κ = 0.80 or higher.

**Findings:**

Participants described two main sources of EVD-related stress: isolation from the community because of social distancing and other prevention measures such as quarantine, and stigma related to infected or affected status. Participants linked experiences of social isolation and stigma to significant distress and feelings of ostracization. These experiences were particularly pronounced among children. Sources of support included community reintegration over time, and formal community efforts to provide education and establish protection bylaws.

**Interpretation:**

This study found that social distancing and EVD-related stigma were each prominent sources of distress among participants. These results suggest that isolation because of infection, and the enduring stigmatization of infected individuals and their families, demand coordinated responses to prevent and mitigate additional psychosocial harm. Such responses should include close engagement with community leaders to combat misinformation and promote community reintegration.

## Introduction

The 2013–2016 Ebola virus disease (EVD) epidemic in West Africa resulted in more infections and deaths than all prior outbreaks combined. Approximately 21% of the 28,000 people infected were children under the age of 16 [1]. The greatest number of confirmed cases were in Sierra Leone, with 41% of these cases in Western Urban and Western Rural, and another 26% of cases in the provincial districts of Port Loko, Kailahun, and Kenema [2].

Studies on psychosocial implications of infectious disease have largely focused on chronic conditions such as HIV/AIDS, commonly associated with stigma [3]. Those infected with or exposed to infectious diseases such as Zika, H1N1, and EVD experience high rates of stigmatization [4, 5] including workplace discrimination, losing a place to live, feeling ashamed, [6] and disrupted social relationships [7]. In Sub-Saharan Africa, tuberculosis stigma was associated with economic strain, such as food insecurity, as well as mental health challenges, including hopelessness and major depressive episodes [8]. COVID-19 stigma and disrupted community support (due to lockdowns, social distancing, and fear of disease transmission) were associated with post-traumatic stress disorder, anxiety, depression, and insomnia [9].

A meta-analysis of 50 research studies (*n* = 92,722) across infectious diseases (SARS, MERS, Zika, H1N1, Ebola, COVID-19) found a 34% pooled prevalence of stigma, which was higher in low- and middle-income countries (37%) compared to high-income countries (27%) [10]. The method of disease transmission influences disease stigmatization. In the context of HIV/AIDS, already stigmatized individuals (such as men who have sex with men, sex workers, and intravenous drug-users) are at disproportionate risk for HIV/AIDS disease infection [11]. As Zika infection led to increased miscarriage and birth defects, women were most negatively affected by disease stigmatization and their children were born with microcephaly [12]. EVD affects all populations and is spread through contact with bodily fluids, but less is known about how EVD infection—and preventive measures such as social distancing—extend to experiences of stigma and social exclusion. Understanding these issues becomes more important in the context of the COVID-19 pandemic, amidst quarantines and intermittent community lockdowns.

Social distancing, an important facet of mitigating the spread of contact, droplet, and airborne diseases, may also be related to stigmatization. Public misunderstanding of quarantine protocols during the 2003 SARS outbreak in the United States and Canada resulted in stigmatization against at-risk groups [13] and health workers [14]. Similar dynamics emerged during the COVID-19 pandemic, with health workers experiencing social exclusion, hostility and interpersonal violence [15]. Stigma may be motivated by disease avoidance and is reflected in negative interactions with community members post-recovery [16].

Disease-related stigma has been associated with lower testing and treatment, hampering contact tracing [3, 10, 11, 17, 18]. The risks posed by EVD-related stigma are heightened given recent findings that EVD survivors may relapse and infect others [19, 20]. EVD survivors also experience stigma when returning to their communities, manifesting as social avoidance, lost jobs and housing, destruction of personal belongings, and interpersonal violence [21], verbal abuse and healthcare neglect [22]. Stigma from having survived EVD has been associated with decreased mental and physical quality of life [23].

Relatively little literature on stigma related to acute infectious diseases has focused on children and caregivers. Anecdotally, children orphaned by EVD are often neglected by families and shunned by their communities because of fear of contagion [11]. These children experience the dual stigmas of being associated with EVD as well as by orphanhood, with community members being unwilling or unable to care for children orphaned by EVD [15].

The developmental implications of disease-related stigma for children are profound [24] and need further examination to inform future intervention development [25]. Moreover, existing literature does not differentiate between the negative psychological effects of social distancing [10] versus stigmatization. This study is designed to address these gaps by examining the extent to which EVD child and caregiver survivors, and those close to them, experienced quarantine-related distress, or stigma and social exclusion, in the immediate aftermath of infection. The study is guided by two research questions: (1) How, if at all, did experiences of psychological distress differ between isolation due to public health measures such as quarantine and social distancing, versus stigmatization by community members for children and adults? and (2) How, if at all, did these experiences evolve over time during community reintegration?

## Methods

### Participants

Participants were selected using purposive maximum variation sampling to target a range of individuals experiencing EVD [26]. Our sampling frame delineated three categories: those infected with EVD; affected family members or caregivers (those living with an infected person but who were not themselves infected); and community leaders.

Key informant interviews (KIIs; *n* = 42) were conducted in five districts: Western Area Urban, Western Area Rural, Kenema, Kailahun, and Port Loko. Focus group discussions (FGDs; *n* = 27) were held in Kenema, Western Urban, and Port Loko. Study districts were selected for a variety of reasons. The high prevalence of EVD in these communities provided a larger purposive sampling pool of EVD-infected individuals, as well as those in the community who had lived with an infected family member. The geographic spread of the districts ensured representation from around the country, and the varying traditional ethnic groups therein. In order to avoid the appearance of ideological favoritism, representation of the country’s political groups was also considered in our selection process.

Participants were recruited from survivor lists provided by the Sierra Leone Association of Ebola Survivors (SLAES), a local non-governmental organization and key study partner. District-level SLAES community liaisons collaborated with local colleagues to identify key leaders, such as imams, pastors, teachers, nurses, chiefs, NGO representatives, and youth association leaders to participate in FGDs. The final sample consisted of 228 participants, composed of primarily EVD-infected (46%) and -affected (42%) individuals, as well as community leaders (12%). The sample included 52% adults and 48% children and youth (see Table 1).

**Table 1:** Participant characteristics.

	Key-Informant (*n =* 42)	Focus Group (*n =* 187)
Age–M (SD)	19.50 (11.29)	N/A
Group–*n* (%)		
Children	31 (73.8%)	71 (37.8%)
Adults	11 (26.2%)	116 (62.2%)
Status–*n* (%)		
Infected	28 (66.7%)	74 (39.6%)
Affected	14 (33.3%)	86 (46.0%)
Leaders/Elders	N/A	27 (14.4%)
Gender–*n* (%)		
Male	24 (57.1%)	82 (51.3%)
Female	18 (42.9%)	78 (48.8%)

*Gender demographics for focus groups do not include community leaders/elders

### Procedures

Data collection occurred over five weeks (June-July of 2019). Prior to entering communities for data collection, community advisory boards (CABs) were established in each study District. SLAES representatives contacted village chiefs and other key leaders to schedule a meeting that described the study and to obtain their feedback on study questions and procedures. These leaders then sensitized their community members about the purpose and process of data collection.

All data collectors were Sierra Leoneans with previous experience as research assistants trained in interview techniques, group facilitation skills, and risk of harm and child protection protocols. In the event of participant emotional distress, SLAES community liaisons and a social worker assessed the need for mental health referrals. During data collection, the team also provided medical referrals for 18 EVD survivor participants (12 adults). The most common symptoms included ocular problems (*n* = 5), joint pain (*n* = 5), sexual dysfunction (*n* = 4), menstrual problems (*n =* 2), and headaches/migraines (*n =* 3).

As part of our duty of care, if any participant disclosed a health issue during the course of data collection, the research assistant made note of the issue and privately asked the participant following the interview whether they had been able to seek treatment, and whether they would like to speak to a doctor. If they answered that they would like to speak to a doctor, the research assistant would obtain consent to share their information with the lead research assistant. They would then work to facilitate having the participant seen immediately at a local medical center, at a time convenient to the participant, where they could receive appropriate attention from a medical professional. For child participants, parents were asked for their consent prior to making any referrals. In all cases, the team was able to facilitate medical referrals shortly after data collection.

KIIs with EVD-infected and -affected participants explored how EVD-related stressors affected family functioning, parent-child and community relationships. Questions included topics such as community relationships during and after the EVD outbreak (e.g. “How, if at all, did Ebola affect your relationships in the community when the epidemic was at its worst?” “How, if at all, has Ebola affected your relationships in the community now that the epidemic is over?”), sources of stress (e.g. “Since Ebola, what are your greatest sources of stress, if any, as a caregiver?”), sources of support (e.g. “Thinking of these sources of stress, what is most helpful to you in managing these stressors?” “Since Ebola, what people in your life, if any, help you most with these problems?”), and community acceptance of EVD survivors (e.g. “How has the community accepted or not accepted Ebola survivors as they resumed normal activities? What about survivors’ families and children?”). Questions varied by type of respondent.

FGDs were each composed of between three and ten participants. Focus groups were single-gender and included: EVD-affected parents/caregivers; EVD-affected children; EVD-infected parents/caregivers; EVD-infected children; and mixed-gender elder/leader groups. Duration of interviews varied based on the age of the interviewee and interview type, but typically ranged from 1–1.5 hours.

Adult participants were paid the equivalent of USD $5.00 in Leones for participation. Children were given household items (i.e. rice, cooking oil, soap) worth the same amount. All participants were provided transportation costs and refreshments. In addition to participant interviews, summaries and field notes were collected following every data collection on perceptions and interactions with participants. These additional materials assisted in the contextualization and interpretation of findings.

All KIIs and FGDs were conducted in Sierra Leonean Krio, Mende, or Temne. Recordings were forward translated by the Caritas research team first into Krio, and then back translated into English and transcribed following protocols established by the World Health Organization [27]. Transcriptions were reviewed by the project manager and research assistants for accuracy.

Prior to entering the field, all research assistants were trained in the meaning and importance of consent and assent. During training, the team practiced administering consent/assent forms in Krio, Mende, and Temne. Given low community literacy rates, all consent and assent forms were read aloud to participants by their assigned research assistant, after which the participant would provide an oral consent/assent. Paper copies were provided in English and Krio, the two primary written languages in Sierra Leone, to all participants who wanted one.

For child participants, caregivers joined interviews for the consent/assent process only. The caregiver was consented first, and if this was agreed to, the child was assented. After this process was complete, the caregiver would leave the interview. A written “X” as a confirmation of oral consent was collected electronically in REDCap. Ethical approvals were granted by the Boston College and Tulane University IRBs, as well as the Sierra Leone Ethics and Scientific Review Committee.

### Data analysis

We used a grounded theory approach and a strategy derived from Thematic Content Analysis for qualitative data analysis [28, 29]. Grounded theory is appropriate for a systematic analysis of qualitative data, using an inductive approach to analysis that builds an understanding of participants’ lived experience, rather than a deductive, theory driven approach [28]. Thematic analysis was also used to identify and organize patterns within the data, using an inductive approach to identify themes strongly linked to the data themselves rather than driven by a theoretical framework [29]. For the purposes of this study, we opted to provide a detailed account of specific themes related to our research questions, rather than provide a full rich description of the entire dataset [29].

A team of researchers from Boston College and partner organizations in Sierra Leone jointly open coded interview transcripts, initially meeting as a large group to establish consensus on preliminary codes (e.g. stigma, community reintegration, etc.) as the team coded transcripts line by line. A smaller analysis team continued to meet to code transcripts until the team reached a saturation point. At this stage, the team conducted axial coding to build thematic structures such as “EVD-related stressors” like mocking, discrimination, or provocation, and “EVD-related supports,” including instrumental support, food sharing, and community protection bylaws. Boston College researchers coded the remaining transcripts together and independently after finalizing the codebook. Training and reliability testing continued until all independent coders reached a Cohen’s kappa at κ = 0.80 or higher using NVivo [30].

## Results

Inductive analyses identified three overarching themes related to our questions in this study: EVD-related stressors, such as social distancing and stigmatization; and EVD-related supports such as community reintegration, community education efforts, and community protection bylaws.

### EVD-related stressors

Most participants described negative experiences with social distancing or EVD-related stigma. We have differentiated between social distancing as a disease prevention method, versus stigmatization by community members.

### Social distancing as a source of distress

Social distancing was among the most commonly cited sources of distress during the epidemic (see Table 2). Codes of community fear of transmission and physical distancing among infected individuals were slightly more frequent among adults (*KII* = 81.8%, *FGD* = 100%) than children (*KII* = 77.4%, *FGD* = 90%). Social distancing typically manifested as community members refusing to come near infected participants or to share items such as food or dishes. One woman, a caregiver infected by EVD, recounted in a KII:

*“My neighbors when I take something because we used to share things in common, if I take anything from them they will say no don’t take this anymore let us don’t share things in common. When you get well we will share things in common but for now let us don’t share things in common.”*

**Table 2:** EVD-related stressors and supports.

	Key Informants (*n %*)			Focus Groups (n %*)		
	Children	Adults	Total	Children	Adults	Total
**EVD-related stressors**						
**Social distancing**	22 (71.0%)	9 (81.8%)	31 (74.8%)	9 (90.0%)	15 (100%)	24 (96.0%)
**Stigma**	24 (77.4%)	10 (90.9%)	34 (81.0%)	10 (100%)	15 (100%)	25 (100%)
Rejection/pushing far	19 (61.3%)	8 (72.7%)	27 (64.3%)	7 (70.0%)	15 (100%)	22 (88.0%)
Mocking/provocation/ bullying	15 (48.4%)	2 (18.2%)	17 (40.5%)	4 (40.0%)	12 (80.0%)	16 (64.0%)
Associative stigma	11 (35.5%)	4 (36.4%)	15 (35.7%)	5 (50.0%)	10 (66.7%)	15 (60.0%)
Discrimination	8 (25.8%)	4 (36.4%)	12 (28.6%)	3 (30.0%)	8 (53.3%)	11 (44.0%)
Judgement/pointing fingers	2 (6.5%)	4 (36.4%)	6 (14.3%)	1 (10.0%)	6 (40.0%)	7 (28.0%)
**EVD-related supports**						
Community reintegration	21 (67.7%)	10 (91.0%)	31 (73.8%)	10 (100%)	15 (100%)	25 (100%)
Community protection bylaws	2 (6.9%)	5 (45%)	7 (16.7%)	2 (20.0%)	5 (33.3%)	7 (28.0%)
Educational Efforts	4 (13%)	7 (64%)	11 (26.1%)	2 (20.0%)	7 (46.7%)	9 (36.0%)

*Percentages indicate the proportion of focus groups mentioning each topic

Another woman, a caregiver affected by EVD, shared in an FGD:

*“So when they quarantined us I felt it, you were not to go to another person’s place to beg for water or for any other things they will tell you to go and sit at your house.”*

Although social distancing and quarantine were necessary measures to prevent or limit the spread of EVD, many participants reported feelings of distress and ostracization. A youth participant said of their experience:

*“When I was infected they were saying don’t come close to me, don’t touch me, I began to cry.”*

Some participants used the term ‘stigmatization’ to describe the experience of quarantine. One adult in focus group said:

*“I was infected, even the toilet that we were sharing they avoid using it, even to give me food they come and put it far from me and walk away, they were stigmatizing me so I was feeling lonely.”*

Physical distancing and other forms of social isolation experienced after recovery, or by individuals who were never infected, were categorized as stigmatization. This phenomenon, in which those connected to infected individuals also experienced social exclusion, is discussed further in the stigmatization subsection below. Additionally, negative experiences reported during infection or quarantine such as bullying or violence were considered stigma.

### Social exclusion and stigmatization

Approximately 93% of participants reported experiencing one or more types of stigmatization related to EVD. Though the likelihood of reporting any stigmatization was similar between children and adults, children reported more frequent experiences of stigma in their communities during and after the outbreak.

The most common form of stigma cited by both child (*KII* = 61.3%, *FGD* = 70%) and adult (*KII* = 72.7%, *FGD* = 100%) participants was rejection or “pushing far.” Participant experiences of rejection post-EVD were often similar to physical distancing measures taken during EVD. Ongoing fear of transmission appeared to be the primary motivation for this form of stigma. One child, a girl infected with EVD, recounted in a FGD:

*“After we’ve been discharged from the hospital and returned home, I saw all my mates playing. I don’t want to go there, if I go and join my friends, they will say I should stop following them, that I should not give them Ebola.”*

Sequelae among survivors led to fear of ongoing infection. A community leader stated:

*“When they came back in the community it was not easy for them, people were thinking that the sickness was not over yet because they were having signs and symptoms, some were having headaches, eye problems, some were deaf, so because of those things they were thinking that the virus is still in them.”*

The second most frequently cited type of stigmatization was mocking, bullying, and violence, frequently referred to by participants as provocation. This usually involved being teased or laughed at for their status as an Ebola survivor or for the status of a family member, and was more commonly experienced by child key informants (*KII* = 48.4%) than adults (*KII =* 18.2%). Though rare, several participants reported experiencing physical violence from their peers (*n* = 3). When asked about his relationship with the family members who lived in his home, a child who was affected by EVD recounted in an FGD:

*Like for me*, *we were not in a good relationship*. *It was two apartments*, *some are upstairs and others downstairs*. *When the children who are downstairs come upstairs to us they will beat them up and drive them to go down stairs for them not to bring the sickness to them*.

Associative stigma was also common among both children and caregivers (*KII* = 35.7%, *FGD* = 60%) and refers to exclusion experienced by those close to infected individuals. Similar to phenomena observed during previous infectious disease outbreaks [13–16] personal connection with infected individuals sometimes led to stigmatizing those who were themselves never infected with EVD. One survivor described how his spouse was treated after he was discharged from a treatment center:

*“When she went back to work her boss with another colleague was afraid of touching her, they said your husband was infected you so don’t come and give us trouble here.”*

A number of children (*KII* = 25.8%, *FGD* = 30%) and adults (*KII* = 36.4%, *FGD* = 53.3%) reported experiencing discrimination, including being denied entry and service at markets and religious institutions. A woman, a caregiver infected by Ebola, discussed her experiences with discrimination in one KII:

*“I used to go to mosque but after I was discharged they were not allowing us to enter the mosque and even some of my friends were also not allowed to enter the church.”*

The least frequent category of stigma among children (*KII* = 6.5%, *FGD* = 10%) and adults (*KII* = 36.4%, *FGD* = 40%) was judgment or community members “pointing fingers” at them. One female caregiver discussed in an FGD in reference to her husband who had EVD:

*“If they pass by and see me they will point fingers at me saying that my husband was the one that brought the Ebola that killed our families.”*

### EVD-related supports

Participants identified a number of post-EVD supports. Some of these occurred organically through a process of community reintegration, and others were more intentional such as community education efforts and community protection bylaws.

### Community reintegration

Despite common reports of social stigma, some of which continued to persist, community reintegration was reported by approximately 89% of participants, and was cited as a major source of support post-EVD. One woman, an EVD survivor, described her newly harmonious relationship with her community members in a KII:

*We mingle, we eat together, talk together and take part in community activities. We join organizations just like in the past. There was a misunderstanding between us before, but since the time community mobilizers intervene and make peace between us, we are living happily now*.

The support that community reintegration provided was described by both adults (KII = 91%, FGD = 100%) and children (*KII* = 67.7%, *FGD* = 100%). Children described reintegration as a cessation of the stigmatization from their peers. One child who survived EVD said,

*I have some friends that were laughing at me when I was sick with Ebola…they don’t point fingers at me anymore. We all live as one now*.

### Community education efforts

Reintegration into social groups and communities typically occurred over time, and was attributed by many adults (*KII* = 64%, *FGD* = 46.7%) and children (*KII* = 13%, *FGD* = 20%) to education efforts from community leaders, healthcare workers, and NGO workers. These educational efforts aimed to combat misinformation and to decrease stigmatization of survivors and their families. In one focus group, a village chief described the role that local and religious leaders played in reintegrating people into the community through messages of unity and familial bonds. He said:

*We were disseminating messages in the community that the people were living with us until they got sick. This message went from counselor to counselor, from headman to headman, from Imam to Pastor, Pastors preach it in the churches, the Imam’s preach it in the mosque- that those people are our families and let nobody point fingers at them because of the stigma*.

Health care workers and NGOs conducted sensitization programs to help people understand when people were no longer contagious, which decreased some of the stigma experienced through fears of disease transmission. In one focus group, an NGO worker explained how educating about EVD helped combat stigmatization:

*People said that it was the lack understanding about Ebola sickness that caused stigma. Some NGOs and other organizations came to help them, and that was the time people were welcomed in their communities and the people accepted them again. Also, the health workers came in to treat them, and they also came in with adult education in the community. That was the time some people started welcoming [survivors]*.

### Community protection bylaws

To a lesser extent, adults (*KII* = 45%, *FGD* = 28%) and some children (*KII* = 6.9%, *FGD* = 20%) describe the effectiveness of community protection bylaws established to decrease the spread of disease and penalize the stigmatization of survivors. One woman, a caregiver who had been infected, reflected during a focus group about a specific bylaw that punished provocation of survivors and their families with monetary fines:

*The community people made a bylaw, saying anyone who provoked us has to pay five hundred thousand leones (Le 500,000). Even for me, people in my area rejected me. I got Ebola at home when I was working in the hospital. My friends who I used to do things with stopped coming to see me. But we thank God now, all what that was happening to us has reduced a lot now. We are as one now, but it was not easy..”*

Although most participants reintegrated into their communities, approximately 11% reported either personally experiencing continued stigma amongst their peers, or witnessing persistent community stigma particularly among children. One child, a boy infected by Ebola, stated in a KII:

*“I was talking to one boy because it was him who was provoking me anytime I went to the field. I told two of them, ‘I am not infected with the sick anymore, stop provoking me. I don’t like your habits. I have told you to stop, the sickness is over, I have been treated please stop.’ That is what I was telling them…they are still persisting.”*

Community leaders expressed uncertainty about whether recovered individuals are still contagious, sometimes related to ongoing physical sequelae. One community leader shared in an FGD:

*“People stigmatized those children because anybody that had Ebola you can see the sign and symptoms on him/her that this person has been infected before. So you see they were thinking that those signs like headache, eye problems means the virus is still in them if we touch those people the virus will transfer to us. It is not really easy for them.”*

## Discussion

The results of this study show that those infected or otherwise affected by EVD experienced significant distress related to social distancing as well as from community stigma. Many participants seemed to equate quarantine with stigmatization by the community, and at least one other EVD study in Sierra Leone also found that quarantined individuals perceived higher levels of community stigma [31]. This finding reflects the dynamics of physical and social avoidance of those affected by infectious disease, but typically this avoidance lessons upon remission of symptoms [10]. Our study confirms that EVD survivors continue to experience stigma so long as physical sequelae such as eye problems and headaches continue. This situation is further complicated by recent findings that EVD remains in survivors’ bodily fluids and can reinfect them [12, 13]. In terms of survivors’ experiences, however, the distress of experiencing disease avoidance from social distancing appears equal to the distress of community stigmatization–and that disease avoidance can in fact function as a type of stigmatization for survivors [10].

Most participants affected by EVD reintegrated into their communities successfully over time [31, 32] linked to intentional efforts at the community level to support survivors as well as to informal processes of trust-building within the community. At three years post-outbreak, the passage of time appears to be a factor in rebuilding relationships that had been disrupted by EVD. Yet, community efforts during and after the outbreak were also important in battling misinformation about Ebola. These intentional efforts by community members to disseminate accurate information were identified as being crucial to supporting the reintegration of EVD survivors. Importantly, community leaders such as pastors and imams were engaged as partners to reach the broader community. These efforts were supplemented with more top-down approaches such as the implementation of community by-laws to reduce stigmatization. What is clear, however, is that intentional engagement of community leaders was a key factor in counteracting misinformation and promoting survivor reintegration efforts.

The persistent stigma experienced by children post-infection is of particular concern, given the significant developmental consequences of stigmatization [18]. The impact of stigmatization for children exposed to infectious diseases is unknown and warrants further research. In our sample, stigmatization did not appear to be linked exclusively to physical sequelae, but rather to being labeled as an EVD survivor more than three years since the outbreak. Similar experiences were documented in Sierra Leone pertaining to child soldiers involved in the country’s civil conflict [33]. As in the war-related sensitization campaigns during that period, children EVD survivors should be a focus of stigma-reduction initiatives which are adjusted and adapted to needs over time.

A recent review of stigma-reduction interventions outside of Sierra Leone found few focused on children but that school-based educational approaches are most commonly implemented [25]. The most effective approaches target multiple levels of the social ecology, given that stigma occurs at individual, interpersonal, institutional and social levels [25, 34]. Such strategies include combinations of information-based approaches, facilitating contact between those affected and not affected, engaging opinion leaders, peer counseling, and skill building interventions [35].

### Limitations

This study has limitations. Given that three years passed between the end of the epidemic in 2016 and data collection in 2019, the reliability of participants’ recalled experiences during EVD warrants consideration, particularly among children. However, a primary aim of this study was to understand how experiences of social exclusion and stigmatization evolved over time during the process of community reintegration following the end of the epidemic, a process which often spanned a significant period of time and is, for some, still ongoing. Another study limitation is the potential bias introduced by a study team led by U.S.-based researchers. The team made efforts to coordinate closely with Sierra Leone-based researchers in developing the initial codebook and interpreting findings. Yet, the positionality of U.S.-based researchers may have influenced the development and interpretation of codes and themes that miss the nuances of local cultural understanding.

## Conclusion

The results of this study suggest that isolation- and stigma-related distress from infectious diseases poses a public health concern beyond the infection itself [7]—and risks becoming a “parallel pandemic” alongside infectious disease outbreaks [36]. Individuals suffering from an infectious disease may avoid testing and treatment out of fear of isolation and stigma, thus exacerbating their own symptoms and increasing risks to public health. Prevention methods such as quarantine are critical in mitigating the spread of highly infectious diseases. Yet, these methods often come with unintended consequences, such as experiencing stigma during quarantine that may persist even after the recovery period. Policy makers, medical personnel, public health officials and social workers must be aware of these unintended negative effects and be prepared to support individuals and communities after periods of isolation. The COVID-19 pandemic and re-emerging cases of EVD and other high-threat pathogens [37] demand coordinated responses to outbreaks and stigma alike–and community leaders are key actors in helping respond to both.

## Supporting information

S1 Questionnaire (DOCX)

## References

[1] World Health Organization (2021). Ebola virus disease fact sheet. https://www.who.int/news-room/fact-sheets/detail/ebola-virus-disease

[2] Richards P., Mokuwa G. A., Vandi A., Mayhew S. H., & Ebola Gbalo Research Team. (2020). Re-analysing Ebola spread in Sierra Leone: the importance of local social dynamics. PloS one, 15(11), e0234823. doi: 10.1371/journal.pone.0234823 33151945 PMC7644078

[3] Parker R., & Aggleton P. (2003). HIV and AIDS-related stigma and discrimination: A conceptual framework and implications for action. Social Science & Medicine, 57, 13–24. 12753813 10.1016/s0277-9536(02)00304-0

[4] Fischer L., Mansergh G., Lynch J., & Santibanez S. (2019). Addressing disease-related stigma during infectious disease outbreaks. Disaster Medicine and Public Health Preparedness, 13, 989–994. doi: 10.1017/dmp.2018.157 31156079 PMC6889068

[5] Mak W., Poon C., Pun L., & Cheung S. F. (2007). Meta-analysis of stigma and mental health. Social Science & Medicine, 65(2), 245–261. doi: 10.1016/j.socscimed.2007.03.015 17462800

[6] Simbayi L. C., Strebel A., Cloete A., et al. (2007). Internalized stigma, discrimination, and depression among men and women living with HIV/AIDS in Cape Town, South Africa. Social Science & Medicine, 64(9), 1823–1831. 17337318 10.1016/j.socscimed.2007.01.006 PMC4271649

[7] Hosek S. G., Harper G. W., Lemos D., & Martinez J. (2008). An ecological model of stressors experienced by youth newly diagnosed with HIV. Journal of HIV/AIDS Prevention in Children & Youth, 9(2), 192–218. doi: 10.1080/15538340902824118 20216916 PMC2834206

[8] Naidu T., Pillay S. R., Ramlall S., Mthembu S. S., Padayatchi N., Burns J. K., et al (2020). Major depression and stigma among individuals with multidrug-resistant tuberculosis in South Africa. The American Journal of Tropical Medicine and Hygiene, 103(3), 1067. doi: 10.4269/ajtmh.19-0426 32700662 PMC7470525

[9] Semo B. W., & Frissa S. M. (2020). The mental health impact of the COVID-19 pandemic: implications for sub-Saharan Africa. Psychology research and behavior management, 13, 713. doi: 10.2147/PRBM.S264286 32982500 PMC7508558

[10] Yuan K., Huang XL., Yan W. et al. A systematic review and meta-analysis on the prevalence of stigma in infectious diseases, including COVID-19: a call to action. Mol Psychiatry (2021). doi: 10.1038/s41380-021-01295-8 34580416 PMC8475479

[11] Davtyan M., Brown B., & Folayan M. O. (2014). Addressing Ebola-related stigma: lessons learned from HIV/AIDS. Global health action, 7(1), 26058. doi: 10.3402/gha.v7.26058 25382685 PMC4225220

[12] Marbán-Castro E., Villén-Gonzalvo A., Enguita-Fernàndez C., Marín-Cos A., Menéndez C., Maixenchs M., et al. (2020). Uncertainties, fear and stigma: perceptions of Zika virus among pregnant women in Spain. International Journal of Environmental Research and Public Health, 17(18), 6643. doi: 10.3390/ijerph17186643 32933007 PMC7559627

[13] Person B., Sy F., Holton K, Govert B., Liang A., and the NCID/SARS Community Outreach Team. (2004). Fear and stigma: The epidemic within the SARS outbreak. Emerging Infectious Diseases,10(2), 358–363. doi: 10.3201/eid1002.030750 15030713 PMC3322940

[14] Digiovanni C., Conley J., Chiu D., & Zaborski J. (2004). Factors influencing compliance with quarantine in Toronto during the 2003 SARS outbreak. Biosecurity and Bioterrorism: Biodefense Strategy, Practice, and Science, 2(4), 265–272. doi: 10.1089/bsp.2004.2.265 15650436

[15] Trejos-Herrera A. M., Vinaccia S., & Bahamón M. J. (2020). Coronavirus in Colombia: Stigma and quarantine. Journal of Global Health, 10(2): 020372. doi: 10.7189/jogh.10.020372 33110564 PMC7568913

[16] Oaten M, Stevenson RJ, Case TI. Disease avoidance as a functional basis for stigmatization. Philosophical Transactions of the Royal Society B: Biological Sciences. 2011;366(1583):3433–52. doi: 10.1098/rstb.2011.0095 22042920 PMC3189356

[17] Denis-Ramirez E., Sorensen K., & Skovdal M. (2017). In the midst of a ‘perfect storm’: Unpacking the causes and consequences of Ebola-related stigma for children orphaned by Ebola in Sierra Leone. Children and Youth Services Review, 73, 445–453.

[18] Sullivan B. J., Esmaili B. E., & Cunningham C. K. (2017). Barriers to initiating tuberculosis treatment in sub-Saharan Africa: a systematic review focused on children and youth. Global health action, 10(1), 1290317. doi: 10.1080/16549716.2017.1290317 28598771 PMC5496082

[19] Mbala-Kingebeni P., Pratt C., Mutafali-Ruffin M.,… & Muyembi Tamfun J.-J. (2021). Ebola Virus Transmission Initiated by Relapse of Systemic Ebola Virus Disease. New England Journal of Medicine, 384:1240–7. doi: 10.1056/NEJMoa2024670 33789012 PMC7888312

[20] Subissi L. (2018). Can Ebola virus re-emerge from survivors’ body fluids other than semen? The Lancet Infectious Diseases, 18(9), 933–934. doi: 10.1016/S1473-3099(18)30435-3 30049621

[21] Karamouzian M. & Hategekimana C. (2015). Ebola treatment and prevention are not the only battles: Understanding Ebola-related fear and stigma. International Journal of Health Policy Management, 4(1), 55–56. doi: 10.15171/ijhpm.2014.128 25584356 PMC4289040

[22] James P., Wardle J., Steel A., & Adams J. (2020). An assessment of Ebola-related stigma and its association with informal healthcare utilization among Ebola survivors in Sierra Leone: A cross-sectional study. BMC Public Health, 20, 182. 32020858 10.1186/s12889-020-8279-7 PMC7001224

[23] James P. B., Wardle J., Gyasi R. M., Steel A., Adams J., Kabba J. A., et al. (2022). Health-related quality of life among Ebola survivors in Sierra Leone: the role of socio-demographic, health-related and psycho-social factors. Health and Quality of Life Outcomes, 20(1), 1–14. 35033102 10.1186/s12955-022-01916-y PMC8761046

[24] Nayar Usha S., Stangl Anne L., De Zalduondo Barbara & Brady Laura M. (2014). Reducing Stigma and Discrimination to Improve Child Health and Survival in Low- and Middle-Income Countries: Promising Approaches and Implications for Future Research, Journal of Health Communication, 19:sup1, 142–163, doi: 10.1080/10810730.2014.930213 25207451 PMC4205916

[25] Hartog K., Hubbard C. D., Krouwer A. F., Thornicroft G., Kohrt B. A., & Jordans M. J. D. (2020). Stigma reduction interventions for children and adolescents in low- and middle-income countries: Systematic review of intervention strategies. Social Science & Medicine, 246. doi: 10.1016/j.socscimed.2019.112749 31978636 PMC7313083

[26] Creswell J.W. and Plano Clark V.L. (2011) Designing and Conducting Mixed Methods Research. 2nd Edition, Sage Publications, Los Angeles.

[27] Toma G, Guetterman TC, Yaqub T, Talaat N, Fetters MD. A systematic approach for accurate translation of instruments: Experience with translating the Connor–Davidson Resilience Scale into Arabic. Methodological Innovations. July 2017. doi: 10.1177/2059799117741406

[28] Strauss A., & Corbin J. M. (1998). Basics of qualitative research: Techniques and procedures for developing grounded theory (2nd ed.). Thousand Oaks, CA, Sage Publications.

[29] Braun V., & Clarke V. (2006). Using thematic analysis in psychology. Qualitative Research in Psychology, 3(2), 77–101, doi: 10.1191/1478088706qp063oa 32100154

[30] QSR International. (2010). NVivo Qualitative Data Analysis Software version 9. http://www.qsrinternational.com

[31] Davidson M.C., Lu S., Barrie M.B., Freeman A., Mbayoh M., Kamara M., et al. A post-outbreak assessment of exposure proximity and Ebola virus disease-related stigma among community members in Kono District, Sierra Leone: A cross-sectional study, Social Science & Medicine—Mental Health (2022), 10.1016/j.ssmmh.2022.100064. PMC9017820 35449727

[32] Kelly JD, Weiser SD, Wilson B, Cooper JB, Glayweon M, Sneller MC,… (2019). Ebola virus disease-related stigma among survivors declined in Liberia over an 18-month, post-outbreak period: an observational cohort study. PLoS neglected tropical diseases 13 (2), e0007185 doi: 10.1371/journal.pntd.0007185 30811388 PMC6411197

[33] Betancourt T. S., Agnew-Blais J., Gilman S. E., Williams D. R., et al. (2010). Past horrors, present struggles: The role of stigma in the association between war experiences and psychosocial adjustment among former child soldiers in Sierra Leone. Social Science & Medicine, 70(1), 17–26. doi: 10.1016/j.socscimed.2009.09.038 19875215 PMC3756934

[34] Rao Elshafei, Nguyen Hatzenbuehler, Frey Go. A systematic review of multi-level stigma interventions: state of the science and future directions. BMC Med., 17 (1) (2019), p. 41, doi: 10.1186/s12916-018-1244-y 30770756 PMC6377735

[35] van Brakel W.H., Cataldo J., Grover S. et al. Out of the silos: identifying cross-cutting features of health-related stigma to advance measurement and intervention. BMC Med 17, 13 (2019). doi: 10.1186/s12916-018-1245-x 30764817 PMC6376667

[36] Villa S., Jaramillo E., Mangione D., et al. (2020). Stigma at the time of the COVID-19 pandemic. Clinical Microbiology & Infection, 26(11), 1450–1452. doi: 10.1016/j.cmi.2020.08.001 32777361 PMC7411378

[37] Qayum I. (2019). Top ten global health threats for 2019: the WHO list. Journal of Rehman Medical Institute, 5(2), 01–02.

